# Smoking and Secondhand Smoke Exposure at Home Were Associated with Poor Perceived Family Well-Being: Findings of FAMILY Project

**DOI:** 10.1371/journal.pone.0161761

**Published:** 2016-08-25

**Authors:** Xin Wang, Man Ping Wang, Kasisomayajula Viswanath, Alice Wan, Tai Hing Lam, Sophia S. Chan

**Affiliations:** 1 School of Public Health, The University of Hong Kong, Hong Kong SAR, China; 2 School of Nursing, The University of Hong Kong, Hong Kong SAR, China; 3 Center for Community-Based Research, Dana-Farber Cancer Institute/Department of Social and Behavioral Sciences, Harvard School of Public Health, Boston, United States of America; Dartmouth College Geisel School of Medicine, UNITED STATES

## Abstract

**Introduction:**

To investigate the associations of cigarette smoking and secondhand (SHS) exposure at home with family well-being among Chinese adults in Hong Kong.

**Methods:**

Telephone surveys were conducted among 3043 randomly selected adults (response rate 70%) in 2010 and 2012 to monitor family health information and tobacco use in Hong Kong. Family well-being was measured using three questions of perceived family harmony, happiness and health (3Hs) with responses ranging from 0–10 and a higher score indicating better family well-being. Smoking status, nicotine dependence, quitting behaviours and SHS exposure at home were recorded. Multiple linear regressions were used to calculate β-coefficients for individual family 3Hs component and an overall composite score representing family well-being.

**Results:**

Compared with never smokers, current smokers reported lower levels of family harmony (adjusted β = -0.15, 95% CI: -0.35 to -0.10), happiness (adjusted β = -0.12, 95% CI: -0.28 to -0.02), health (adjusted β = -0.15, 95% CI: -0.30 to -0.03) and overall family well-being (adjusted β = -0.17, 95% CI: -0.32 to -0.06). Quit attempt and intention to quit were not associated with family well-being. SHS exposure at home was associated with lower levels of family harmony (adjusted β = -0.17, 95% CI: -0.30 to -0.07), happiness (adjusted β = -0.19, 95% CI: -0.32 to -0.08), health (adjusted β = -0.13, 95% CI: -0.26 to -0.03) and family well-being (adjusted β = -0.19, 95% CI: -0.32 to -0.09).

**Conclusions:**

Smoking and SHS exposure at home were associated with the lower levels of perceived family well-being. Prospective studies are needed to confirm the results.

## Introduction

Family well-being encompasses physical, social, economic and psychological aspects [1] and was usually assessed using pre-specified family resources, functions or needs; the quality of relationship; social, political and cultural context of families [2,3]. Family well-being is an indicator of harmonious society [4, 5] particularly for Chinese, who put strong emphasis and value on family and social harmony as affected by traditional Confucian ethics [6, 7]. Local studies showed that family structure, economic and workload status are main factors that affect family well-being [4, 6]. Less is known about the influence of individual behaviour factors.

Substance abuse and addiction are some of the behaviour factors associated with poor family functioning [8, 9]. Most studies focused on alcohol and drug abuse and less is known for the adverse effects of smoking on family well-being. Smoking and secondhand smoke (SHS) may be associated with poor family functions given the negative impacts of smoking on mental and physical health, and financial burden [10–13]. Our previous study also showed that smoking was associated with lower levels of individual happiness [14]. Smoking was also associated with poor parenting styles and children substances abuse [15, 16], which are closely linked with poor parent-children relationships. Smoking-related family conflicts were commonly reported in smoking families [17, 18]. Smokers also were found to have poor family communication and marital relationship [19].

In Hong Kong, which is among the lowest smoking prevalence in the developed world, the cigarette smoking prevalence has halved from 23.3% in 1982 to 10.7% in 2013 [20, 21]. Comprehensive smokefree legislation has banned smoking in all indoor work places and most of indoor public places since 2007. Displacement of smoking into the home was observed in homes of children [22]. Given the adverse effects of tobacco on subjective mental and physical health, the financial burden, family functions and relationship, smoking and SHS at home would likely affect family well-being adversely. We found no such studies, thus we examined the associations of smoking and SHS at home with family 3Hs (health, happiness and harmony) and well-being among Chinese adults in Hong Kong.

## Methods

### Ethical Statement

Ethical approval was granted by Institutional Review Board (IRB) of the University of Hong Kong/Hospital Authority Hong Kong West Cluster. As this is a telephone survey, written consent is not possible. Verbal informed consents were obtained and recoded verbatim, and the procedure was approved by the IRB.

### Survey design

Data of the Hong Kong Family and Health Information Trends Survey (FHInTs), which was under the FAMILY project, a Hong Kong Jockey Club Initiative for a Harmonious Society, from 2010 and 2012 were analyzed. The survey, details described elsewhere [14, 23], is a representative telephone survey of Hong Kong adults during 2010/11 (Dec-Mar) and 2012 (Aug-Oct) on general public’s behaviours and opinions related to family harmony, happiness, health (3Hs) and family communications. The telephone interviews were conducted by trained interviewers of Public Opinion Programme (POP) at the University of Hong Kong, which is a locally well-known survey agency. Telephone numbers were first selected randomly from telephone directories as seed numbers. Another set of numbers were then generated by using the plus/minus one/two method. Duplicate numbers were then screened out, and the remaining numbers were mixed in a random order to become the final sample. Upon successful contact with a target household, one eligible member of the household was selected among those present using the ‘next birthday’ rule. Ethical approval was granted by the institutional review board of the University of Hong Kong/Hospital Authority Hong Kong West Cluster.

### Measurements

Our previous qualitative study showed that family harmony, happiness and health are core elements of family well-being in Hong Kong [4, 6]. Based on this, we measured family well-being 3Hs using three separate questions of: “Do you think your family is harmonious/happy/healthy?” with responses ranging from 0 to 10 points with a higher score indicating better family well-being. High internal consistency was observed for these three questions (Cronbach’s alpha 0.89) and 2-week test-retest reliability was 0.81. A composite score of family well-being was calculated by summing up the scores of these three questions. This composite well-being score is significantly correlated with daily average family communication (r = 0.62, p<0.001) and family relationship (r = 0.50, p<0.05). Cigarette smoking status was categorised as current smokers (daily or occasional smoking), ex-smokers, and never smokers. Among current smokers, the number of cigarettes smoked per day, quit attempt during last 12 months and intention to quit were also assessed. Intention to quit was categorised as contemplation (intent to quit within 6 months) or preparation (intent to quit within 1 month) and pre-contemplation (intent to quit after 6 months or no intention) [14]. SHS exposure at home was measured by one question: ‘In general, are you exposed to secondhand smoking at home’. Information on age, gender, education, marital status, employment and income were also collected.

### Statistical analysis

Mean family 3Hs and well-being scores were calculated overall and by age groups (18–24, 25–44, 45–64 and ≥65 years), gender, education, marital status, employment, family income and smoking behaviour. Differences in family 3Hs and well-being scores across sub-groups were tested using Student’s t-test or ANOVA. Crude and adjusted association between family 3Hs and well-being scores with smoking status and SHS exposure at home were examined using multivariate regression models by adjusting for surveyed year and demographic factors including age, gender, education, marital status, employment and family income. For current smokers, the associations between family 3Hs and smoking behaviours (number of cigarette per day, quit attempt in past 12 months and intention to quit) were also examined. Interaction terms between smoking status and SHS exposure at home on family 3Hs and well-being was investigated using multivariate regression model. Stratified analyses were conducted for significant interactions. The overall sample was weighted by age and sex from the Hong Kong census data in the year of survey. All analyses were done using SAS 9.2 (SAS Incorporated, Cary, NC) and a p-value < 0.05 was considered statistically significant.

## Results

Among 4347 confirmed eligible adults, 3043 (70.0%) successfully completed the interviews. As described in previous study, the sample is representative and the survey subjects were similar to Hong Kong 2011 census population data (Cohen’s effects were small 0.02–0.17) [23]. Of all respondents, over half were women (62%), married (66%) and about half were aged 45 to 64 (48%). Over two-thirds had secondary education or above (70%) and nearly half had monthly family income above medium income of general population (HK$20,000) [24].

The mean score of family health, happiness, harmony and well-being was 7.70 (±1.6), 7.42 (±1.6), 7.27 (±1.7) and 22.4 (±4.3), respectively. Older age and higher household income were significantly associated with higher level of family harmony, happiness, health and well-being (all p<0.01). Similar levels of family 3Hs and well-being were observed for gender, education and employment status (Table 1).

**Table 1:** Family harmony, happiness and health by socio-demographic characteristics and cigarette smoking status (N = 3043).

	N (%)	N (%) (weighted ^b^)	Family harmony (1 item, 0–10) (mean 7.70±1.6)		Family happiness (1 item, 0–10) (mean 7.42±1.6)		Family health (1 item, 0–10) (mean 7.27±1.7)		Family well-being (3 items, 0–30) (mean 22.4±4.3)	
			mean (SD)	*p*^a^	mean (SD)	*p*^a^	mean (SD)	*p*^a^	mean (SD)	*p*^a^
Sex				0.69		0.71		0.58		0.83
Male	1162(38.2)	1393(45.8)	7.74(1.6)		7.42(1.7)		7.32(1.7)		22.48(4.4)	
Female	1881(61.8)	1650(54.2)	7.72(1.6)		7.44(1.6)		7.28(1.7)		22.45(4.3)	
Age				<0.001		<0.001		<0.01		<0.001
18–24	345(11.4)	314(10.4)	7.32(1.6)		7.13(1.6)		7.10(1.5)		21.54(4.3)	
25–44	700(23.1)	1124(37.1)	7.57(1.5)		7.39(1.5)		7.15(1.6)		22.11(4.1)	
45–64	1448(47.8)	1114(36.8)	7.82(1.6)		7.49(1.6)		7.37(1.7)		22.70(4.3)	
65+	535(17.7)	476(15.7)	7.96(1.9)		7.55(2.0)		7.40(2.0)		22.91(4.8)	
Marital status				<0.001		<0.001		<0.001		<0.001
Single	796(26.2)	972(32)	7.29(1.6)		7.07(1.6)		6.94(1.6)		21.30(4.3)	
Married/cohabitated	2009(66.1)	1881(61.9)	7.95(1.6)		7.63(1.6)		7.47(1.7)		23.05(4.1)	
Others (divorced/widowed)	234(7.7)	186(6.1)	7.42(1.9)		6.99(2.1)		7.03(2.1)		21.39(5.1)	
Education				0.97		0.48		0.45		0.66
≤Primary	582(19.2)	446(14.7)	7.74(2)		7.41(2.0)		7.31(2.1)		22.47(5.2)	
Secondary	1507(49.6)	1435(47.3)	7.72(1.6)		7.41(1.6)		7.26(1.7)		22.4(4.2)	
≥Tertiary	947(31.2)	1155(38)	7.73(1.5)		7.49(1.5)		7.35(1.5)		22.56(4)	
Employment status				0.42		0.77		0.58		0.52
Full-time	1159(38.1)	1422(46.7)	7.69(1.5)		7.42(1.4)		7.29(1.5)		22.41(3.9)	
Part-time	208(6.8)	194(6.4)	7.63(1.5)		7.33(1.5)		7.18(1.7)		22.14(4.1)	
Self-employed	100(3.3)	121(4.0)	7.81(1.7)		7.47(1.7)		7.47(1.7)		22.82(4.3)	
Unemployed	1576(51.8)	1306(42.9)	7.77(1.8)		7.45(1.8)		7.31(1.9)		22.52(4.7)	
Household income, HK$ (US $1 = HK $7.8)				<0.001		<0.001		<0.001		<0.001
<$10,000	565(21.8)	475(18.2)	7.65(1.9)		7.23(2.0)		7.11(2.1)		22.00(5.1)	
$10,000-$19,999	605(23.4)	590(22.6)	7.49(1.7)		7.26(1.6)		7.10(1.7)		21.85(4.4)	
$20,000-$29,999	542(20.9)	561(21.5)	7.80(1.5)		7.47(1.4)		7.36(1.6)		22.65(3.9)	
$30,000-$39,999	344(13.3)	383(14.6)	7.91(1.4)		7.56(1.4)		7.52(1.5)		22.99(3.7)	
≥$40,000	535(20.7)	606(23.2)	7.95(1.4)		7.74(1.3)		7.51(1.4)		23.20(3.7)	
Cigarette smoking				0.005		0.18		0.07		0.02
Never-smokers	2587(85.2)	2550(83.9)	7.74(1.6)		7.44(1.6)		7.31(1.7)		22.50(4.3)	
Ex-smokers	207(6.8)	208(6.8)	7.92(1.6)		7.53(1.8)		7.42(1.9)		22.86(4.4)	
Current smokers	244(8.0)	280(9.2)	7.44(1.8)		7.26(1.7)		7.07(1.8)		21.77(4.6)	
Daily cigarette consumption (mean ± std)				0.65		0.65		0.34		0.73
Daily smokers (n = 151)	15.8 (6.7)	15.6 (7.5)	7.40 (1.8)		7.30 (1.7)		6.99 (1.8)		21.69 (4.5)	
Occasional smokers (n = 94)	3.6 (2.1)	3.4 (2.1)	7.51 (1.8)		7.20 (1.7)		7.22 (1.8)		21.90 (4.7)	
Quit attempt in past 12 months				0.64		0.16		0.99		0.69
Yes	87 (35.5)	101 (36.1)	7.53 (1.7)		7.06 (1.8)		7.08 (1.8)		21.63(4.6)	
No	158 (64.5)	180 (63.9)	7.41 (1.9)		7.38 (1.6)		7.08 (1.8)		21.88(4.5)	
Intention to quit ^c^				0.53		0.29		0.76		0.55
Contemplation or preparation	49 (20.2)	56 (20.3)	7.29 (2.0)		7.04 (2.0)		7.00 (1.9)		21.42(5.2)	
Pre-contemplation	193 (79.8)	221 (79.7)	7.48 (1.8)		7.33 (1.6)		7.09 (1.8)		21.86(4.4)	
SHS at home				<0.001		<0.001		<0.001		<0.001
Yes	381 (12.6)	365 (12.0)	7.36 (1.8)		7.02 (1.7)		6.95 (1.7)		21.30(4.4)	
No	2656 (87.4)	2673 (88.0)	7.72 (1.6)		7.42 (1.6)		7.29 (1.7)		22.44(4.3)	

^a^ *p*-values based on t-test (2 groups) and ANOVA (3 or 4 groups)

^b^ weighted by age and sex from census data

^c^ Pre-contemplation: intention to quit after 6 months or no intention to quit;

Contemplation: intention to quit after 1 month and within 6 months; Preparation: intent to quit within 1 month.

Table 1 also shows that 8.0% of the sample were current smokers and 35.5% of the cigarette smokers had ever attempted to quit in the past 12 months. About one in ten (12.6%) respondents was exposed to SHS at home. Ex-smokers reported the highest level (22.86±4.4) of family well-being while current smokers had the lowest scores (21.77±4.6) (p = 0.02). For both smokers and non-smokers, those who were exposed to SHS at home reported lower levels of family 3Hs and well-being, compared with those who were not exposed to SHS at home (p<0.001).

Compared to never smoking, current smoking was significantly associated with lower levels of family harmony (adjusted β = -0.15, 95% CI: -0.35 to -0.10), happiness (adjusted β = -0.12, 95% CI: -0.28 to -0.02), health (adjusted β = -0.15, 95% CI: -0.30 to -0.03) and overall well-being (adjusted β = -0.17, 95% CI: -0.32 to -0.06). SHS exposure at home was associated with lower levels of family harmony (adjusted β = -0.17, 95% CI: -0.30 to -0.07), happiness (adjusted β = -0.19, 95% CI: -0.32 to -0.08), health (adjusted β = -0.13, 95% CI: -0.26 to -0.03) and well-being (adjusted β = -0.19, 95% CI: -0.32 to -0.09) (Table 2).

**Table 2:** Association of family harmony, happiness and health with cigarette smoking and exposure to second hand smoke exposure at home (β-coefficients).

	Family harmony (1 item, 0–10)		Family happiness (1 item, 0–10)		Family health (1 item, 0–10)		Family well-being (3 items, 0–30)	
	Adjusted ^a^	*p*	Adjusted ^a^	*p*	Adjusted^a^	*p*	Adjusted^a^	*p*
Sex								
Male	-0.04	0.31	-0.08	0.08	0.01	0.86	-0.04	0.34
Female	0	-	0	-	0	-	0	-
Age								
18–24	-0.20*	0.04	-0.04	0.68	0.11	0.28	-0.05	0.62
25–44	-0.21**	<0.01	-0.07	0.34	-0.05	0.51	-0.13	0.10
45–64	-0.16*	0.02	-0.09	0.18	-0.04	0.52	-0.11	0.11
65+	0	-	0	-	0	-	0	-
Marital status								
Married/cohabitated	0.37***	<0.001	0.38***	< .0001	0.38***	<0.001	0.43***	<0.001
Others (divorced/widowed)	-0.04	0.71	-0.11	0.27	0.11	0.26	-0.03	0.80
Single	0	-	0	-	0	-	0	-
Education								
≤Primary	-0.05	0.51	-0.07	0.31	-0.07	0.32	-0.07	0.31
Secondary	-0.07	0.16	-0.08	0.09	-0.08	0.08	-0.09	0.06
≥Tertiary	0	-	0	-	0	-	0	-
Employment status								
Full-time	-0.02	0.69	-0.02	0.64	0.00	0.93	-0.02	0.76
Part-time	-0.03	0.74	-0.06	0.49	-0.03	0.70	-0.04	0.61
Self-employed	-0.01	0.88	-0.05	0.62	-0.02	0.88	-0.01	0.92
Unemployed	0	-	0	-	0	-	0	-
Household income, HK$ (US $1 = HK $7.8)								
<$10,000	-0.18*	0.01	-0.25***	<0.001	-0.20**	<0.01	-0.23**	<0.01
$10,000-$19,999	-0.20**	<0.01	-0.19**	<0.01	-0.14*	0.03	-0.20**	<0.01
$20,000-$29,999	-0.04	0.54	-0.10	0.08	-0.06	0.34	-0.07	0.23
$30,000-$39,999	0.04	0.52	-0.04	0.52	0.04	0.58	0.02	0.81
≥$40,000	0	-	0	-	0	-	0	-
Cigarette smoking status								
Current smokers	-0.15*	0.03	-0.12	0.07	-0.15*	0.03	-0.17*	0.02
Ex-smokers	0.03	0.68	0.02	0.78	0.02	0.80	0.02	0.76
Never-smokers	0	-	0	-	0	-	0	-
SHS at home								
Yes	-0.17**	<0.01	-0.19**	<0.01	-0.13*	0.03	-0.19**	<0.01
No	0	-	0	-	0	-	0	-

^a^ Variables were adjusted by year and mutually adjusted.

*p<0.05

** *p* <0.01

*** *p* <0.001.

For smokers, the numbers of cigarettes consumed were significantly associated with lower family well-being (adjusted β = -0.03, 95% CI: -0.06 to -0.003) among daily smokers. However such association was not significant in occasional smokers. Quit attempt in the past 12 months and intention to quit smoking were not associated with family 3Hs and well-being (Table 3).

**Table 3:** Associations of cigarette smoking with family harmony, happiness and health (β-coefficients).

	Family harmony		Family happiness		Family health		Family well-being	
	Crude	Adjusted^a^	Crude	Adjusted^a^	Crude	Adjusted^a^	Crude	Adjusted^a^
Cigarette consumption								
Occasional smokers (n = 94)	-0.02	-0.06	-0.05	-0.05	-0.10	-0.12	-0.06	-0.10
Daily smokers (n = 151)	-0.02	-0.03	-0.03*	-0.03	-0.02	-0.03	-0.03*	-0.03*
Quit attempt in past 12 months								
Yes	0.06	-0.04	-0.22	-0.23	-0.02	-0.04	-0.07	-0.13
No	0	0	0	0	0	0	0	0
Intention to quit ^b^								
Contemplation or preparation	-0.04	-0.08	-0.07	-0.04	0.06	0.09	0.005	0.01
Pre-contemplation	0	0	0	0	0	0	0	0

^a^ Adjusting for year, sex, age, marital, education, employment and income

^b^ Pre-contemplation: intention to quit after 6 months or no intention to quit

Contemplation: intention to quit after 1 month and within 6 months; Preparation: intent to quit within 1 month.

*p<0.05

** *p* <0.01

*** *p* <0.001.

Subgroup analysis by smoking status found strongest associations (adjusted β) among never smokers who were exposed SHS at home (family harmony: -0.17, 95% CI: -0.30 to -0.04; family happiness: -0.17, 95% CI: 0.30 to -0.05; family health: -0.08, 95% CI: -0.22 to 0.05 and wellbeing: -0.16, 95% CI: -0.29 to -0.03) (Table 4). In contrast, non-significant associations were observed for current smokers (p = 0.01 for the interaction term for smoking on associations of SHS exposure with family 3Hs and well-being).

**Table 4:** Association of family harmony, happiness and health with second hand smoke exposure at home (β-coefficients).

	Exposed to SHS at home n (%)	Family Harmony		Family happiness		Family health		Family well-being	
		Crude	Adjusted^a^	Crude	Adjusted^a^	Crude	Adjusted^a^	Crude	Adjusted^a^
Current smokers	43 (24.9)	-0.07	-0.23	0.02	-0.02	-0.27	-0.35	-0.12	-0.23
Ex-smokers	22 (13.6)	-0.44	-0.14	-0.84***	-0.72**	-0.52*	-0.33	-0.69**	-0.46
Never-smokers	314 (16.5)	-0.24***	-0.17*	-0.23***	-0.17**	-0.17**	-0.08	-0.25***	-0.16*

^a^ Adjusting for year, sex, age, marital, education, employment and income

p = 0.01 for the interaction term of SHS and smoking status from multivariate model

*p<0.05

** *p* <0.01

*** *p* <0.001.

Compared with no SHS exposure at home

## Discussion

Our finding showed that current cigarette smoking was associated with poor level of family 3Hs and overall well-being among Chinese adults in Hong Kong. For daily smokers, the increased numbers of cigarette consumption were dose-responsively associated with lower family wellbeing. In contrast, ex-smokers had similar family 3Hs and wellbeing levels compared with never smokers supported the reversibility of smoking cessation on improving family well-being. Smoking is associated with family conflict particularly in Hong Kong where smoking is banned in most public area and home becomes a main place for smoking. Homes in Hong Kong are typically crowded, so smoking affects a majority of non-smoking family members who know the harms of SHS. In fact, quitting smoking is regarded as a responsible behaviour to protect family, particularly in Chinese society.

Smokers had lower socioeconomic status (SES) in most developed countries including Hong Kong. Families with low SES are at risk of poor family functioning due to financial constraints, inability to recognize family problems and a lack of skills and motivation for solving problems [4]. Tobacco tax and the price of cigarettes have increased in recently years as a method to reduce cigarette consumption in Hong Kong [25]. The price of a pack of cigarettes has been raised from HK$39 (US$ 5) in 2009 to HK$54 (US$ 7) in 2014. For a smoker who consumes one pack daily, the expenditure accounts for 8% of a median income family [24]. The economic burden from the increased cigarette cost can cause insufficient financial resources, which will lower family satisfaction and happiness from non-smoking family members, not to mention the direct and indirect morbidity costs due to smoking [26].

Negative association between SHS at home and family wellbeing were also observed, with the strongest association found among non-smokers. It suggests that family smoking and SHS exposure were linked to poor family wellbeing, which indicated the adverse effects of SHS on the individual have been expanded to family as a whole. Family conflict is a key feature of disharmony [4] and smoking related conflict is one of the commonly reported disharmony by couples particularly if only one smoked [27], which is common in Hong Kong. The smoking conflict is particularly obvious if children are suffering from SHS exposure from smoking parents. Parental conflict can also cause emotional insecurity in children (e.g. fear and avoidance), which has been shown to be a robust intervening process in the prospective links between inter-parental conflict and child maladjustment [28]. Such family problems may undermine family harmony and well-being. Current smoking cessation interventions mostly focus on the health issues caused by smoking and SHS. The negative impact on family well-being can be further warned in smoking cessation interventions and smokefree home promotions.

This study has some limitations. First, three single questions on family 3Hs were asked to measure family well-being. Results showed being older, married and wealthy were associated with the greater composite family well-being scores, which are consistent with other studies on other family well-being indicators [2]. However, further studies are needed to confirm the validity of these 3 questions on family 3Hs. Second, the concept of family well-being may be different between Chinese and Western countries. Future culture specific or comparative studies may be warranted. Third, the cross-sectional nature of this study precludes the causal inference, as well as the temporality or direction of the associations. Although the notion of reverse causality of poor family wellbeing on smoking and SHS exposure was less likely, prospective studies are needed to confirm the findings.
